# Supplementary material for: Deregulated Myt3 translation predisposes islet β-cells to dysfunction under obesity-induced metabolic stress
Source: bioRxiv. 2025 May 15:2025.05.11.653323. Preprint. [Version 1] doi: 10.1101/2025.05.11.653323 (PMC12132573; doi:10.1101/2025.05.11.653323)
Supplement: Supplement 1 [file media-1.pdf]

Summary of Supplementary Materials:

1) Materials and Methods.
2) Supplementary table description (1 table).
3) Supplementary Figures and legend (three figures).

Materials and Methods:

IPGTT, GISS assays, RNA-seq data analysis, and IF analysis utilized methods described in the manuscript.

Supplementary Table S1. Spreadsheet for gene expression analysis.

Supplementary Figures:

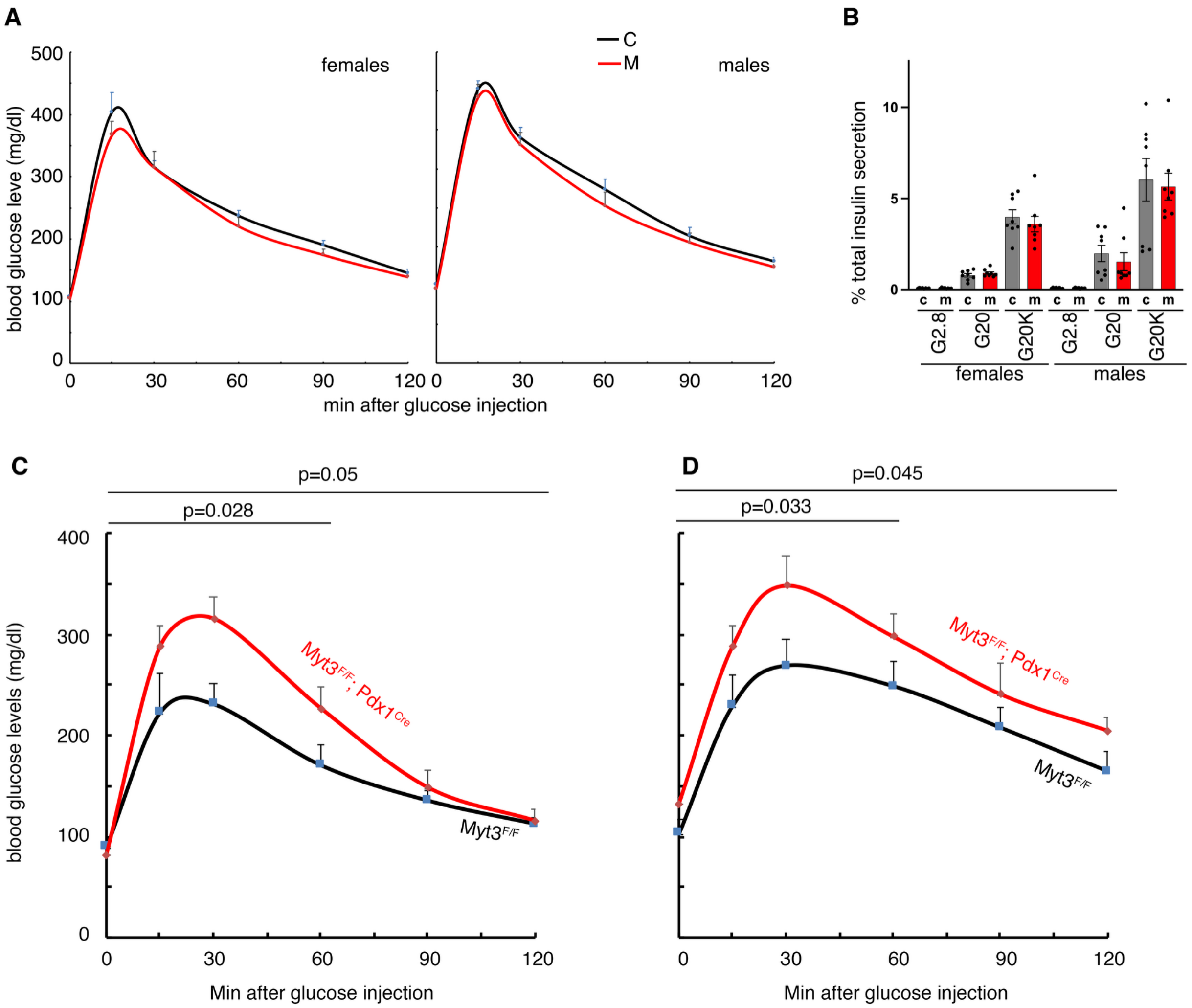

Figure S1. Myt3 is required for glucose homeostasis in aged mice. (A) IPGTT of 8-week-old mice. In both sexes, 6 controls and 6 mutants were tested. (B) Islet GSIS of 8-week-old mice, shown as % of total insulin secreted within a 45-minute window. Each assay contained at least 4 mice, 2-3 technical repeats from each mouse. (C, D) IPGTT of ~5-month-old mice. In females, 6 controls and 6 mutants were tested. In males, 6 controls and 7 mutants were tested.

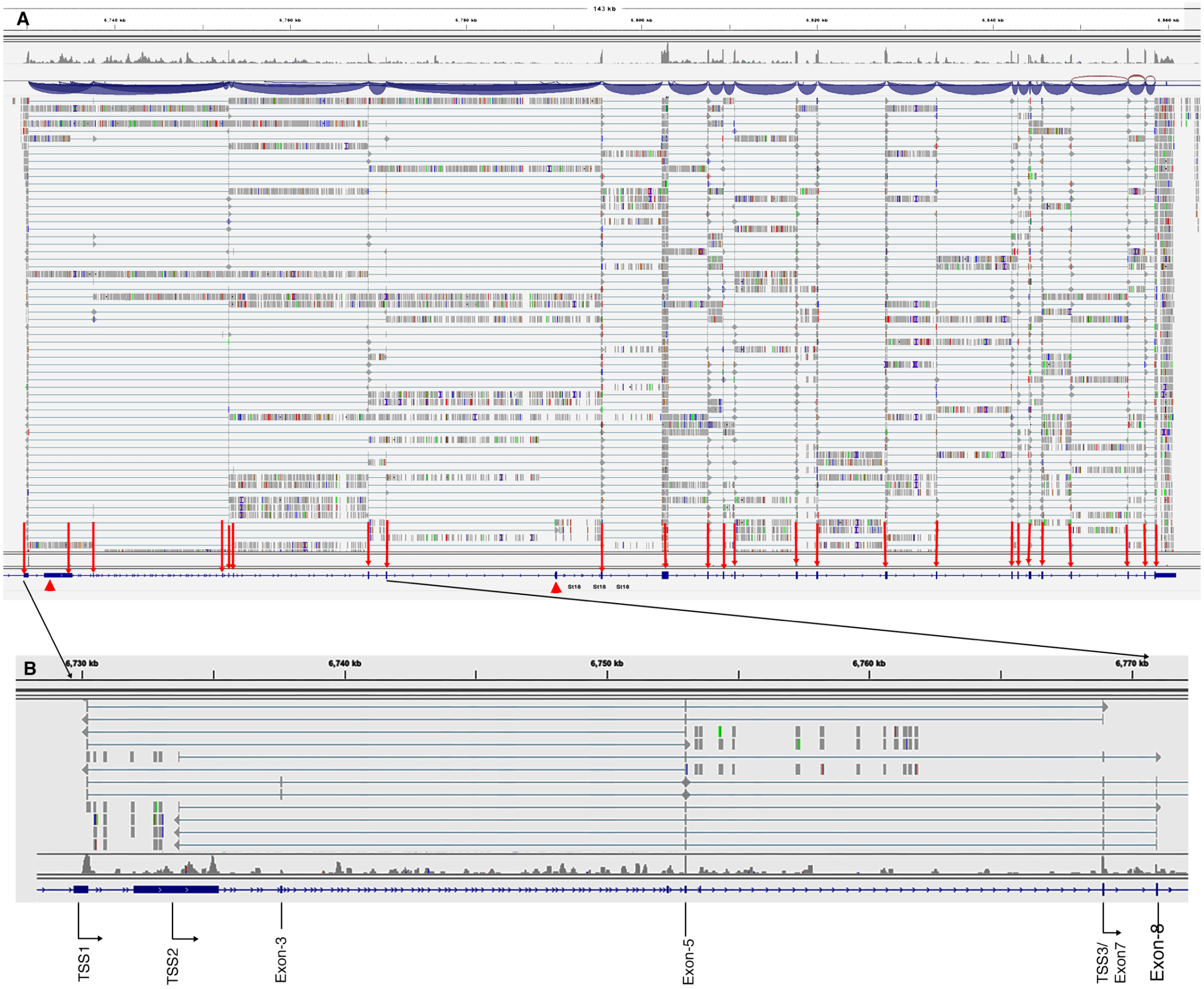

Figure S2. Exon expression of Myt3 in purified adult β cells. The original RNAseq data will be posted upon publication.

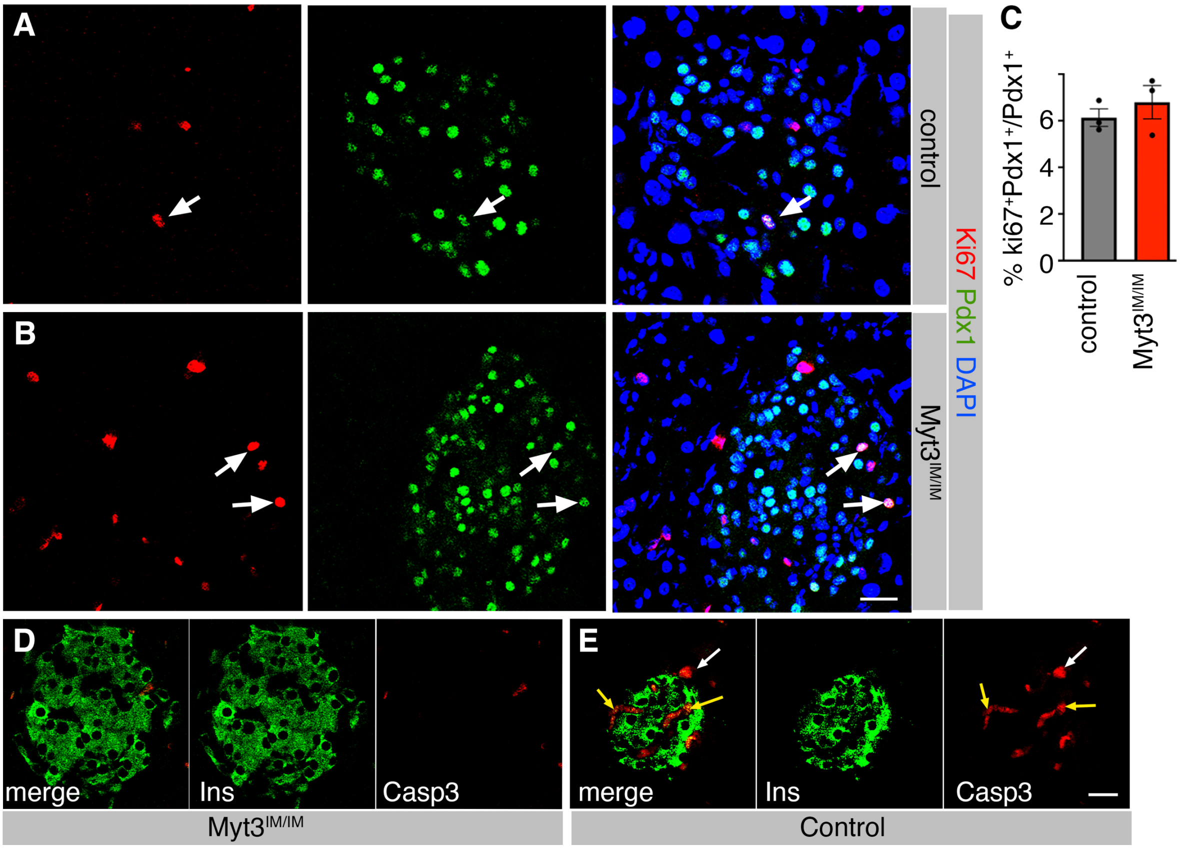

Figure S3. Myt3$^{IM/IM}$ cells have normal proliferation and viability under HFD treatment. (A-C) Ki67 and Pdx1 staining in control and Myt3$^{IM/IM}$ islet cells after 3-month feeding with HFD. The white arrow, a Ki67^{+}Pdx1^{+} cell, appears to be dividing. (D, E) Apoptosis assays (via cleaved caspase 3, Casp3) in β cells of control and Myt3$^{IM/IM}$ islet cells after 3-month feeding with HFD. White arrows, a Casp3^{+}Ins^{-} cell, to show that the IF assay was working. Yellow arrows, blood cells, recognized by their shape and location within the islets. Bars, 20 μm.
